# Polygenic Prediction of Recurrent Events After Early-Onset Myocardial Infarction

**DOI:** 10.1161/CIRCGEN.124.004687

**Published:** 2024-11-29

**Authors:** Maddalena Ardissino, Elvezia Maria Paraboschi, Samuel A. Lambert, Lois G. Kim, Martin Kelemen, Giuseppe Maglietta, Antonio Crocamo, Giulia Magnani, Serena Bricoli, Luigi Vignali, Giampaolo Niccoli, Marco Tubaro, Libor Pastika, Arunashis Sau, Fu Siong Ng, Antonio de Marvao, Michael C. Honigberg, Pradeep Natarajan, Adam J. Nelson, Michael Inouye, Emanuele Di Angelantonio, Rosanna Asselta, Diego Ardissino, Adam S. Butterworth

**Affiliations:** British Heart Foundation Cardiovascular Epidemiology Unit, Department of Public Health and Primary Care (M.A.,S.A.L., L.G.K., M.K., M.I., E.D.A., A.S.B.).; Victor Phillip Dahdaleh Heart and Lung Research Institute (M.A.,S.A.L., L.G.K., M.K., M.I., E.D.A., A.S.B.).; Cambridge Baker Systems Genomics Initiative, Department of Public Health and Primary Care (S.A.L., M.I.).; Health Data Research UK Cambridge, Wellcome Genome Campus (S.A.L., M.I., E.D.A., A.S.B.).; National Institute for Health and Care Research Blood and Transplant Research Unit in Donor Health and Behaviour (L.G.K., E.D.A., A.S.B.).; British Heart Foundation Centre of Research Excellence, School of Clinical Medicine, University of Cambridge, United Kingdom (M.I., A.S.B.).; National Heart and Lung Research Institute (M.A.,L.P., A.S., F.S.N.).; Medical Research Council-London Institute of Medical Sciences, Imperial College London, United Kingdom (M.A.,A.M.).; Department of Biomedical Sciences, Humanitas University (E.M.P., R.A.).; IRCCS Humanitas Research Hospital, Milan, Italy (E.M.P., R.A.).; Clinical and Epidemiological Research Unit (G. Maglietta), University Hospital of Parma.; Department of Cardiology (A.C., G. Magnani, S.B., L.V., G.N.), University Hospital of Parma.; Department of Medicine and Surgery, University of Parma (D.A.).; Division of Cardiology, San Filippo Neri Hospital, Rome, Italy (M.T.).; Department of Women and Children’s Health (A.M.).; British Heart Foundation Center of Research Excellence, School of Cardiovascular Medicine and Sciences, King’s College London, United Kingdom (A.M., E.D.A.).; Program in Medical and Population Genetics, Broad Institute of Harvard & MIT, Cambridge (M.C.H., P.N.).; Cardiovascular Research Center, Massachusetts General Hospital, Harvard Medical School, Boston (M.C.H., P.N.).; Adelaide Medical School, University of Adelaide (A.J.N.).; Cambridge Baker Systems Genomics Initiative, Baker Heart and Diabetes Institute, Melbourne, Victoria, Australia (M.I.).; Health Data Science Centre, Human Technopole, Milan (E.D.A.).; Associazione per lo Studio della Trombosi in Cardiologia, Pavia, Italy (D.A.).

**Keywords:** cardiovascular diseases, coronary artery disease, genetic risk score, genetics, heart disease risk factors

## Abstract

**BACKGROUND:**

Myocardial infarction (MI) is a complex disease caused by both lifestyle and genetic factors. This study aims to investigate the predictive value of genetic risk, in addition to traditional cardiovascular risk factors, for recurrent events following early-onset MI.

**METHODS:**

The Italian Genetic Study of Early-Onset Myocardial Infarction is a cohort study enrolling patients with MI before 45 years. Monogenic variants causing familial hypercholesterolemia were identified, and a coronary artery disease polygenic score (PGS) was calculated. Ten-fold cross-validated Cox proportional hazards models were fitted sequentially including all clinical variables, the PGS, and monogenic variants on the composite outcome of cardiovascular death, recurrent MI, stroke, or revascularization.

**RESULTS:**

During a 19.9-year follow-up, 847 (50.7%) patients experienced recurrent events. Each 1-SD higher PGS was associated with a 21% higher hazard of recurrent events (hazard ratio, 1.21 [95% CI, 1.13–1.31]; *P*=4.04×10^−6^). Except for secondary prevention, PGS was the strongest determinant of recurrent event risk (C index, 0.56 [95% CI, 0.54–0.58]) compared with clinical risk factors. Overall, predictive performance of clinical risk factors (C index, 0.69 [95% CI, 0.67–0.71]) improved after adding the PGS (C index, 0.69 [95% CI, 0.68–0.71]; *P*=0.006). When dividing the population by PGS quintiles, the highest fifth had a 57% higher hazard of recurrent events than the lowest fifth (hazard ratio, 1.57 [95% CI, 1.26–1.96]; *P*=5.57×10^−5^).

**CONCLUSIONS:**

When compared with other clinical risk factors, PGS was the strongest predictor of event recurrence among patients with an early-onset MI. Though the discriminative power of recurrent event prediction in this cohort was modest, the addition of PGS significantly improved discrimination.

After experiencing a myocardial infarction (MI), a key concern for both patients and clinicians is the risk of further cardiovascular events. This is especially true for patients who experience MI at an early age. In recent years, polygenic scores (PGSs) have been shown to improve prediction of incident cardiovascular events above and beyond traditional risk factors.^1–6^ In recent investigations in the UK Biobank^7^ and in French Canadians,^8^ PGSs were shown to also improve recurrent event prognostication after a first MI in addition to clinical factors, with genetic risk being among the strongest predictors of recurrence. However, in the setting of early-onset MI, where a proportionally greater genetic contribution is recognized,^9,10^ the value of PGSs for recurrent event prediction is unclear.

The Italian Genetic Study of Early-Onset Myocardial Infarction is a longitudinal cohort study enrolling patients with an early-onset MI occurring before the age of 45 years. The aim of the present study is to evaluate the predictive value of polygenic risk in addition to traditional risk factors for prediction of recurrent events after early-onset MI.

## Methods

The original study protocol was approved under 4272/98 Ospedale Niguarda, Ca’ Granda on March 9, 1998 and by the Ethics Committee and Institutional Review Board of the coordinating center. Written informed consent was given by all of the patients. The conditions of ethical approval do not permit public archiving of the anonymized study data. The Methods for this study are available in the Supplemental Material.

## Results

### Population Characteristics

**Figure 1.**
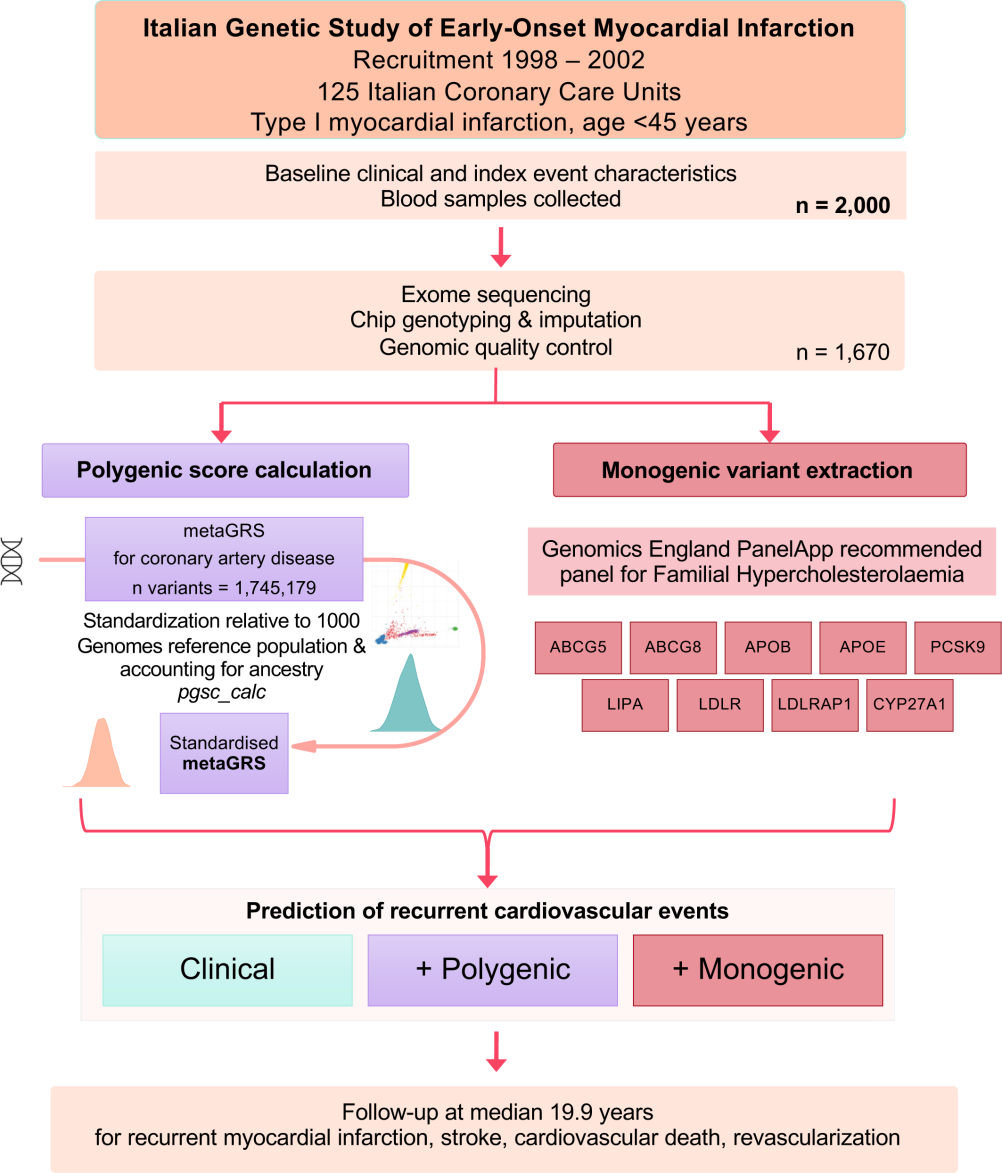
**Study design and data processing.** metaGRS indicates polygenic risk score.

This study included 1670 individuals with a median follow-up of 19.9 years, as summarized in Figure 1. The key characteristics subdivided by PGS category are presented in Table 1. Mean age at index event was 39.58 (SD=4.88) years. Though all individuals were of self-reported European ethnicity, principal component analysis identified a minority of individuals as genetically more similar to Ad-mixed American (9 individuals) and South Asian (1 individual) ancestries. Principal component distributions of participants and their matching to 1000 Genomes participants is displayed in Figure S1.

**Table 1.**
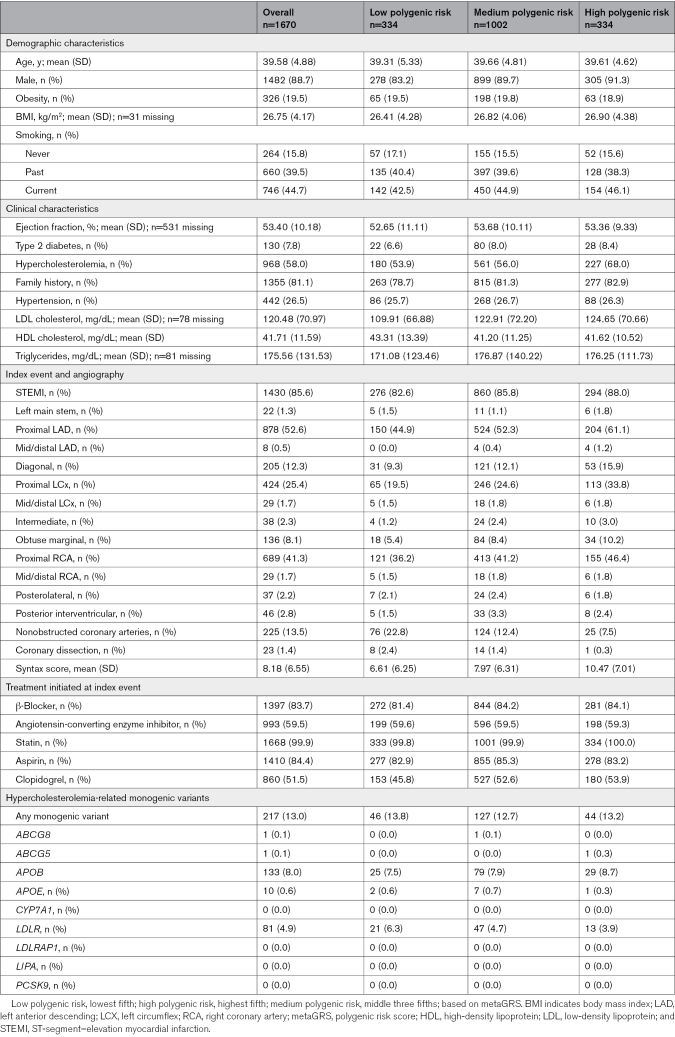
Baseline Demographic and Clinical Characteristics by Polygenic Score Category

The majority of patients had experienced an ST-segment–elevation MI as the index event (85.6%), and this proportion increased across PGS risk categories (82.6% low PGS versus 88.0% high PGS). On angiography, a greater proportion of individuals in the low PGS category had unobstructed coronary arteries (22.8% low PGS versus 7.5% high PGS). Moreover, individuals in the high PGS category tended to have greater coronary atherosclerotic disease burden measured by Syntax score (mean of 10.47 among high PGS versus 6.61 among low PGS).

### Clinical and Genetic Prediction of Risk of Recurrence

**Figure 2.**
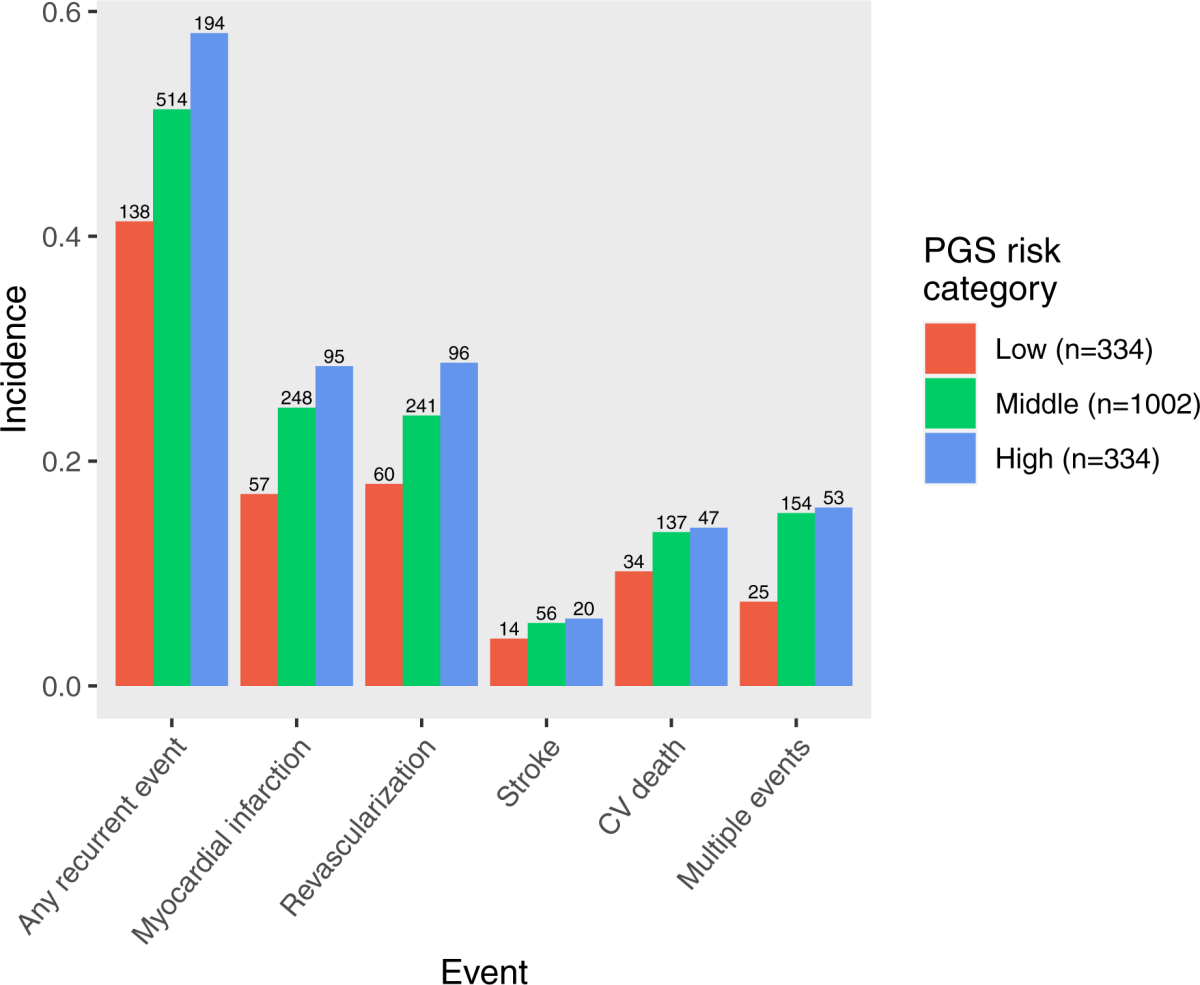
**Incidence of recurrent cardiovascular (CV) events during follow-up among patients with early-onset myocardial infarction with high (top fifth), medium (middle three fifths), and low (bottom fifth) polygenic risk based on metaGRS.** PGS indicates polygenic score; metaGRS, polygenic risk score.

During follow-up, 847 (50.7%) patients experienced at least 1 recurrent event. Mean standardized PGS among those who had a recurrent event was 0.61 (±0.85), compared with 0.40 (±0.86) among those who did not (Figure S2). During follow-up, the incidence of the primary end point was 41.3% in the low PGS group, 51.3% in the middle PGS group, and 58.1% in the high PGS group. Incidence of all cardiovascular events was incremental across increasing PGS categories (Figure 2; Table 2).

**Table 2.**
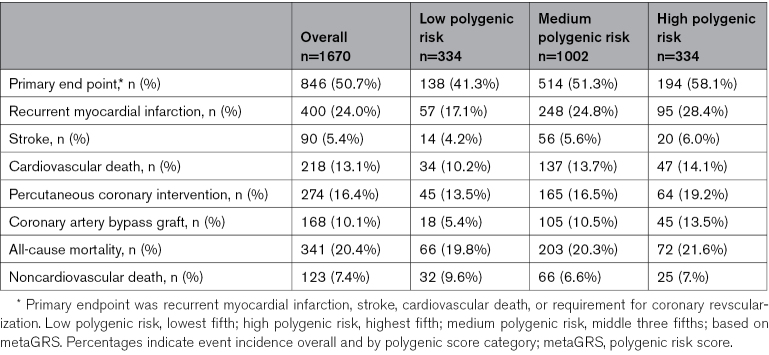
Event Numbers During Follow-Up in Early-Onset Myocardial Infarction Cohort by Polygenic Score Category

**Figure 3.**
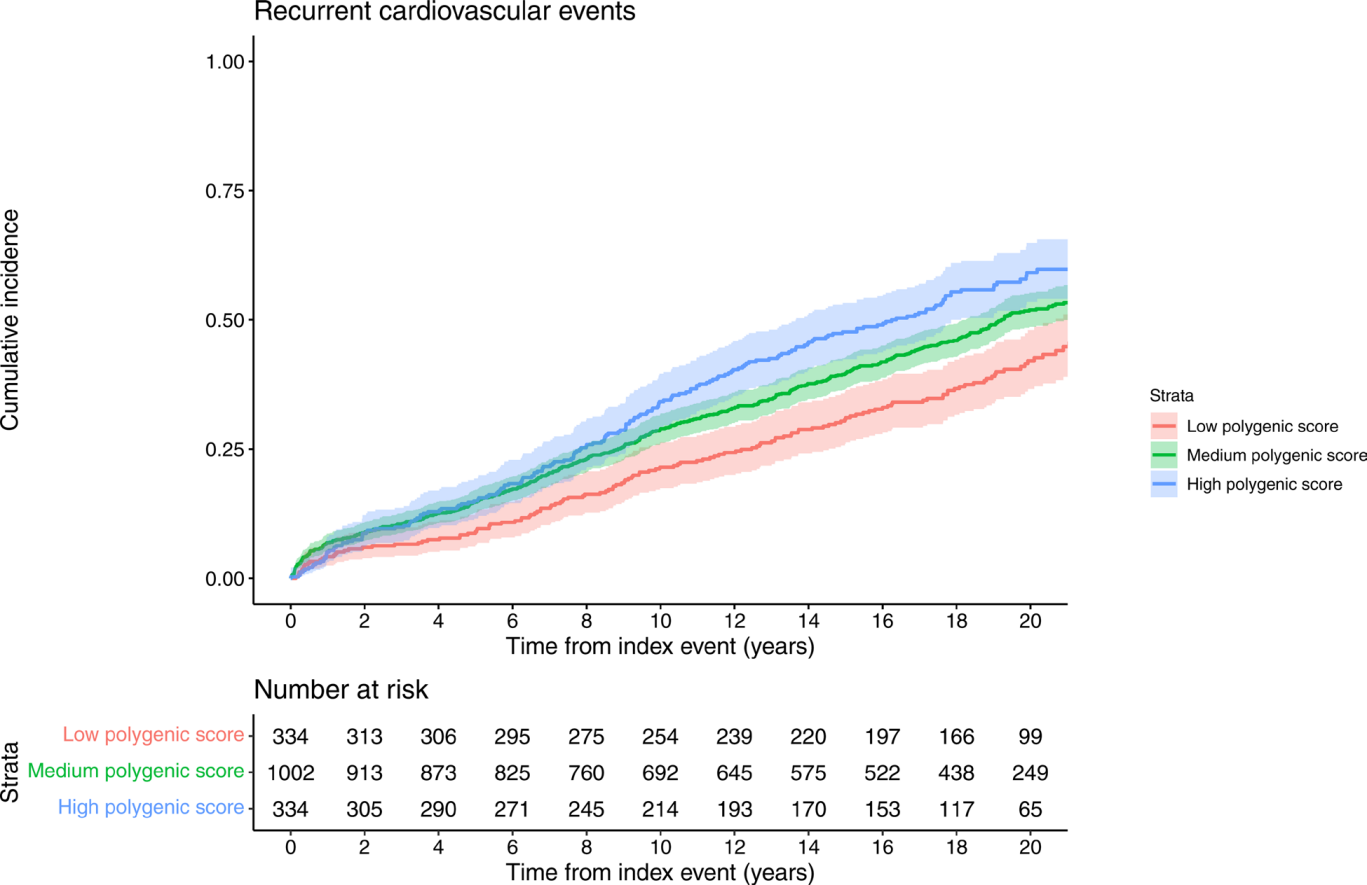
**Cumulative incidence of recurrent cardiovascular events among patients with early-onset myocardial infarction with high (top fifth), medium (middle three fifths), and low (bottom fifth) polygenic risk based on metaGRS.** metaGRS indicates polygenic risk score.

The cumulative incidence of recurrent events by PGS category is depicted in Figure 3. After adjustment for age, sex, secondary prevention medications, and 10 principal components, each 1-SD higher PGS was associated with a 21% higher hazard of recurrent events (hazard ratio, 1.21 [95% CI, 1.13–1.31]; *P*=4.04×10^−6^). Compared with those in the low PGS category, those in the high PGS category had a 57% higher hazard of recurrent events (hazard ratio, 1.57 [95% CI, 1.26–1.96]; *P*=5.57×10^−5^). There was no evidence of violation of proportional hazards assumptions in the tested modes (Schoenfeld residual *P*>0.05).

**Figure 4.**
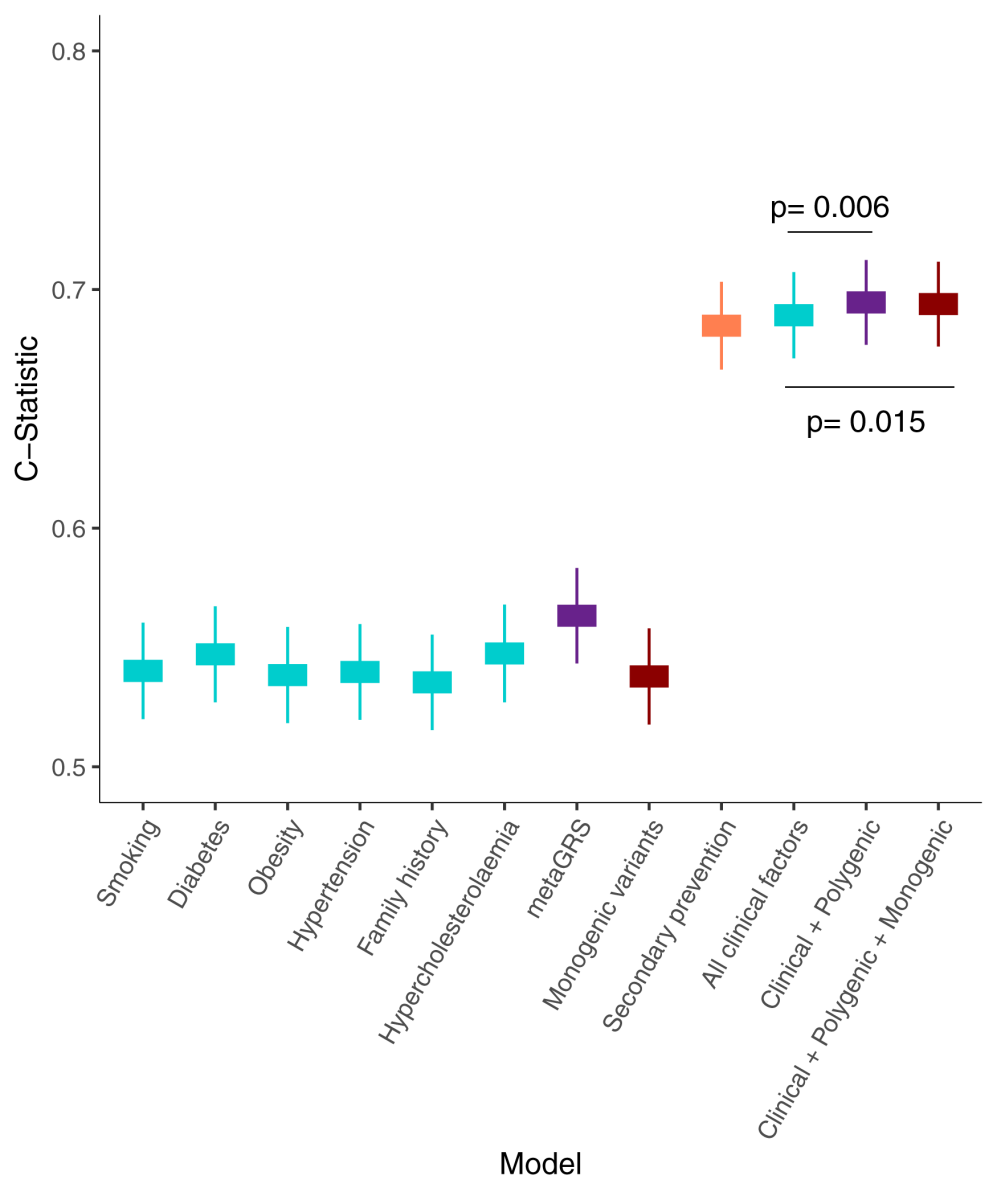
**Discrimination ability of conventional risk factors, secondary prevention, polygenic score (metaGRS), and monogenic variants for recurrent cardiovascular events.** All models include age, sex, and 10 genetic principal components as covariates. metaGRS indicates polygenic risk score.

The discrimination ability of conventional risk factors, PGSs, and monogenic variants for recurrent cardiovascular events is displayed in Figure 4. Compared with smoking (C index, 0.54 [95% CI, 0.52–0.56]), diabetes (C index, 0.55 [95% CI, 0.53–0.57]), obesity (C index, 0.54 [95% CI, 0.52–0.56]), hypertension (C index, 0.54 [95% CI, 0.52–0.56]), family history (C index, 0.54 [95% CI, 0.52–0.56]), and hypercholesterolemia (C index, 0.55 [95% CI, 0.53–0.57]), the PGS alone had the highest predictive power (C index, 0.56 [95% CI, 0.54–0.58]). Use of secondary prevention medications had a high predictive power (C index, 0.68 [95% CI, 0.67–0.70]).

The discriminative ability of the model combining all clinical characteristics (C index, 0.69 [95% CI, 0.67–0.71]) was improved after addition of the PGS (C index, 0.69 [95% CI, 0.68–0.71]; *P* value compared with clinical model, 0.006) and by addition of both the PGS and monogenic variants (C index, 0.69 [95% CI, 0.68–0.71]; *P* value compared with clinical model, 0.015) though addition of the monogenic variants in itself did not appear to sizeably improve discrimination (Figure 4). This result remained consistent across multiple sensitivity analyses, described in the Supplemental Material and Figure S3. Reclassification was also improved after addition of the PGS (continuous net reclassification index compared with clinical model, 0.134 [95% CI, 0.101–0.227]), and a similar improvement was observed after addition of both PGS and monogenic variants (continuous net reclassification index compared with clinical model, 0.150 [95% CI, 0.128–0.260]).

The addition of the PGSs showed an improvement in time-dependent area under the curve (AUC) compared with the model with only clinical factors, which was statistically significant from 12 years after the index event (AUC clinical, 0.71 [95% CI, 0.69–0.74]; AUC clinical+polygenic, 0.72 [95% CI, 0.70–0.75]; *P* value compared with clinical model, 0.006; Figure S4). Similarly, the addition of both PGSs and monogenic variants led to consistently higher estimates for the time-dependent AUCs, which were statistically significant from ≈12 years after the index event (AUC clinical, 0.71 [95% CI, 0.69–0.74]; AUC clinical+polygenic, 0.72 [95% CI, 0.70–0.75]; *P* value compared with clinical model, 0.007; Figure S4).

## Discussion

In this study, the incorporation of genetic information alongside clinical factors enhanced the accuracy of risk stratification for recurrent cardiovascular events following early-onset MI. The results remained consistent in multiple sensitivity analyses. The notion that coronary artery disease risk stratification can be improved by the addition of PGSs to clinical factors has been previously described in multiple primary prevention populations.^1–4,8^ Our results are in line with these previous investigations and extend current knowledge through 3 key findings.

The first and key finding of this study is that PGSs improved discrimination for recurrent events after early-onset MI. In particular, the PGS had the individual strongest discriminative power for recurrent events when compared with clinical factors, a finding that aligns with previous studies. In primary prevention populations, Elliott et al^11^ reported a modest increase in predictive accuracy for prevalent coronary artery disease after addition of PGS to a clinical model, while Inouye et al^2^ similarly reported improved predictive accuracy for incident coronary artery disease. In addition to this, Khera et al^12^ showed that a high PGS was associated with a>3-fold higher odds of early-onset MI. However, all these studies were limited to primary prevention and the detection of first events. In the only presently available observational study on recurrent events, Cho et al^7^ reported that, among individuals with prevalent coronary artery disease in the UK Biobank, age at first coronary artery disease event and elevated PGS were the strongest risk factors for recurrence.

The second finding of this study is that discriminative power of clinical factors for prediction of recurrent events in patients with early-onset MI is limited. Despite the enhancement in discrimination achieved through the incorporation of the PGS, even the best model’s discrimination metrics remained relatively modest. This indicates that a notable proportion of recurrent events remain unexplained, mirroring a similar observation in the recent study by Cho et al^7^ where the combined clinical model achieved a C index of 0.64 (0.63–0.65). Specifically, in our study, conventional clinical factors appeared particularly poorly predictive of recurrent events, and most of the predictive power in the combined models appeared to be derived from the use of secondary prevention. This finding has a clear biological explanation. With a high proportion of patients initiated on secondary prevention medication after the index event,^13^ it might be expected that clinical factors (and potentially, genetic risk) naturally become less strongly predictive of recurrent events. Collectively, in recognizing the relatively limited discriminative efficacy of clinical factors in our specific cohort, our results emphasize the need for tailored risk stratification algorithms specifically catered to patients with early-onset MI, in which PGS might play a role.

The third and final finding involves the interesting observation that the time-dependent Area under the curvemetaGRS - polygenic risk score comparing the clinical model to the clinical+polygenic model progressively diverge over time from the initial event. This trend may stem from the relative stability of PGS as a risk factor compared with other clinical factors. Especially in light of the long follow-up time in this study, some patients might have feasibly developed new risk after the index event, and conversely others with modifiable risk factors at baseline might resolve or control these after the index event. In contrast, the risk that is measured through PGS at the time of index event is not expected to increase or decrease during follow-up, and this stability might grant it better discriminative ability in the long term.

There are a number of limitations to consider. The analysis including monogenic variants could have limited statistical power, leading to suboptimal weighting during the 10-fold cross-validation process. Importantly, there is a lack of standardized methodology for appropriately weighing the potential incremental risk attributed by monogenic variants. Taken together, these factors likely explain the notably small (and in some cases, completely lacking) incremental benefit observed after inclusion of monogenic variants on top of PGSs and clinical variables, though this might also be explained by the risk already being captured in the variable for hypercholesterolemia. On the PGS side, it is important to note that not all PGS variants were available in this cohort (77%) because the genotyping chip used in this population is not recent. Though it must be acknowledged that these missing data might influence results, we expect that the magnitude of potential influence is likely small as variants are likely to be missing at random. In addition to this, although we recruited patients from across Italy, it is essential to acknowledge that this cohort comprises individuals of only self-reported European ethnicity, limiting the generalizability of our findings to populations of other ancestries. Additionally, we do not have data regarding differences in lifestyle and compliance with medications after the index event. Unfortunately, this means that a number of interesting questions cannot be answered. For example, differences in lifestyle and more aggressive management among patients with familial hypercholesterolemia might partly explain why the influence of monogenic variants seems small. Importantly, it must be highlighted that the association estimates of the PGSs with ischemic events from this analysis should not be extrapolated to represent the estimate of association of the PGS with ischemic events overall. This is because this specific population has been selected based on the index event of early-onset MI, and, therefore, the association estimate of the PGSs will be influenced by a degree of index event bias, as recruitment of patients was conditional to an event that is influenced by the PGS to begin with. Related to this, it must also be highlighted that the metaGRS is a score built to evaluate genomic risk of coronary artery disease. Though by definition all patients with early-onset MI have coronary artery disease, early-onset MI is quite a specific phenotype within the umbrella of CAD, and the leading specific genetic drivers of early-onset MI might differ to those of the general (and by tendency, typically older) population with CAD. Because of this, the incremental benefit of PGS might be underestimated in our evaluation.

In conclusion, this study examines the role of PGSs for enhanced prediction of recurrent events among patients with an early-onset MI. The addition of PGS to traditional risk factors significantly improved discrimination. Importantly, PGS alone was the strongest predictor of event recurrence, benchmarking the clinical relevance of this measure for recurrence risk prediction in patients with early-onset MI.

## ARTICLE INFORMATION

### Acknowledgments

The authors acknowledge all investigators and participants who contributed to the Italian Genetic Study of Early-Onset Myocardial Infarction.

### Sources of Funding

This work was supported by the Emilia-Romagna Region: Programma Regione Università Cardiovascular Genetics: from bench to bedside, CUP E35E09000880002 and by core funding from the British Heart Foundation (RG/18/13/33946), National Institute for Health Research (NIHR) Cambridge Biomedical Research Centre (BRC-1215-20014; NIHR203312), Cambridge British Heart Fundation Centre of Research Excellence (RE/18/1/34212), British Heart Fundation Chair Award (CH/12/2/29428), and by Health Data Research UK (Molecules to Health Records programme), which is funded by the Medical Research Council United Kingdom Research Institute, the National Institute for Health Research, the British Heart Foundation, Cancer Research UK, the Economic and Social Research Council United Kingdom Research Institute, the Engineering and Physical Sciences Research Council United Kingdom Research Institute, Health and Care Research Wales, Chief Scientist Office of the Scottish Government Health and Social Care Directorates, and Health and Social Care Research and Development Division (Public Health Agency, Northern Ireland). M. Ardissino is supported by the National Institute for Health Research Academic Clinical Fellowship and Fetal Medicine Foundation (495237). Dr Ng is supported by the British Heart Foundation (RG/F/22/110078 and RE/18/4/34215) and NIHR Imperial Biomedical Research Centre. Dr Natarajan is supported by the US National Heart, Lung, and Blood Institute (National Heart, Lung, and Blood Institute R01HL127564 and NHGRI U01HG011719). Dr Honigberg is supported by the US National Heart, Lung, and Blood Institute (National Heart, Lung, and Blood Institute, K08HL166687) and the American Heart Association (940166 and 979465). Dr Inouye is supported by the Munz Chair of Cardiovascular Prediction and Prevention and the NIHR Cambridge Biomedical Research Centre (BRC-1215-20014; NIHR203312). Dr Inouye was also supported by the UK Economic and Social Research 878 Council (ES/T013192/1). Dr Ardissino was funded by the Associazione per lo Studio della Trombosi in Cardiologia for the Italian Genetic Study of Early-Onset Myocardial Infarction. Dr Butterworth is supported by core funding from the British Heart Foundation (RG/18/13/33946) and NIHR Cambridge Biomedical Research Centre (BRC-1215-20014; NIHR203312). The views expressed are those of the authors and not necessarily those of the NIHR or the Department of Health and Social Care. M. Ardissino was supported by the Medical Research Council Clinical Research Training Fellowship (MR/Z505146/1) grant.

### Disclosures

Dr Natarajan reports research grants from Allelica, Amgen, Apple, Boston Scientific, Genentech/Roche, and Novartis; personal fees from Allelica, Apple, AstraZeneca, Blackstone Life Sciences, Creative Education Concepts, CRISPR Therapeutics, Eli Lilly & Co, Foresite Labs, Genentech/Roche, GV, HeartFlow, Magnet Biomedicine, Merck, and Novartis; scientific advisory board membership of Esperion Therapeutics, Preciseli, and TenSixteen Bio; scientific cofounder of TenSixteen Bio; equity in MyOme, Preciseli, and TenSixteen Bio; and spousal employment at Vertex Pharmaceuticals, all unrelated to the present work. Dr Honigberg reports consulting fees from CRISPR Therapeutics and Comanche Biopharma, advisory board service for Miga Health, and grant support from Genentech, all unrelated to the present work. Dr Inouye is a trustee of the Public Health Genomics Foundation, a member of the Scientific Advisory Board of Open Targets, and has a research collaboration with AstraZeneca PLC, which is unrelated to this study. Dr Butterworth reports institutional grants from AstraZeneca, Bayer, Biogen, BioMarin, Bioverativ, Novartis, Regeneron, and Sanofi. The other authors report no conflicts.

### Supplemental Material

Supplemental Methods

Supplemental Results

Figures S1–S5

References 6,14–32

## Supplementary Material


