# Supplementary material for: Polygenic Prediction of Recurrent Events After Early-Onset Myocardial Infarction
Source: Circ Genom Precis Med. 2024 Nov 29;17(6):e004687. doi: 10.1161/CIRCGEN.124.004687 (PMC11651354; doi:10.1161/CIRCGEN.124.004687)

SUPPLEMENTAL MATERIAL

Supplemental Methods

Patient population

The Italian Genetic Study of Early-Onset Myocardial Infarction patient cohort is a study by "The Atherosclerosis, Thrombosis, and Vascular Biology Italian Study Group" (ATVB)[14]carried out across 125 Italian Coronary Care Units and consisting of an initial case-control study, followed by a prospective study of the cases. Cases and controls were enrolled consecutively between 1998 and 2002.[15] Among the original cohort, 1,686 (84.3%) of the 2,000 cases had available quality-controlled genotyping and sequencing data. Among these, 1,670 (83.5%) had follow-up data available. The study design is summarized in **Figure 1**.

Study protocol

The original study protocol was approved under 4272/98 Ospedale Niguarda, Ca' Granda on 03/09/1998 and by the Ethics Committee of the coordinating centre. Written informed consent was given by all of the patients. After identifying patients suitable for enrolment, investigators completed a standardized case report form collecting detailed cardiovascular history of the individual patients and all first- and second-degree relatives, cardiovascular risk factors, lifestyle, and medications. This was carried out at the time of admission for the index event. The presence and distribution of coronary artery stenosis was established on initial angiography.

Case definition

The cases were eligible if they had been hospitalized because of a first type I myocardial infarction (MI) occurring before the age of 45 years and had undergone

coronary angiography at the time of the index event. The index MI was diagnosed on the basis of a combination of three characteristics:

1) symptoms suggesting myocardial ischemia;
2) Electrocardiographic (ECG) changes consistent with acute myocardial ischemia; and
3) an increase in cardiac biomarker levels (creatine kinase [CK-MB] mass assay and troponin [cTn] T or I assays) to more than twice the upper limit of normal that followed a rise and fall pattern.

Clinical variable definitions

Angiographic coronary artery disease

The presence and distribution of coronary artery stenosis was established on initial angiography. A narrowing of ≥70% (≥50% in the case of the left main coronary artery) was considered significant coronary artery stenosis and documented for each coronary artery and its main branches. The extent and complexity of coronary artery disease were graded by means of the Syntax score, which was computed by means of an online calculator[16]. Angiograms performed at the time of the index event were reviewed by two trained interventional cardiologists. In the case of disagreement, the judgement of a third was integrated, and the final decision was made by consensus.

Family history

A positive family history was defined as the presence of at least one first-degree relative (parent, offspring, or sibling) who developed coronary artery disease before the age of 55 years for men and 65 years for women.

Hypertension

The subjects were considered to have hypertension if they had been diagnosed as

hypertensive or were taking antihypertensive medication.

Obesity

Based on body mass index (BMI) values, patients were categorized as not having obesity (<30 kg/m^2), or having obesity (≥30 kg/m^2).

Smoking habits

The subjects were classified as current, former, or never smokers on the basis of self-reports: current smokers were those who reported smoking regularly during the three years preceding the myocardial infarction; former smokers were those who had smoked regularly for at least three years but not during the year preceding the infarction; and never smokers were those who had never smoked regularly or had smoked regularly for less than three years. Never and former smokers were aggregated in the single category of non-smokers.

Diabetes

The subjects were considered to have diabetes if they had ever been diagnosed as having type I or II diabetes by a physician.

Hypercholesterolemia

Hypercholesterolemia was defined as a fasting total serum cholesterol level of 200 mg/dL or the intake of anti-hypercholesterolemia medications.

Secondary prevention medications

The use of secondary prevention medication was ascertained based on clinical records at the time of discharge. This was coded as binary variables indicating use of aspirin, clopidogrel, beta-blockers, angiotensin-converting enzyme (ACE) inhibitors and statins as separate variables, at the time of discharge. This variable makes no

differentiation between newly initiated versus continued therapy.

Genetic data extraction

DNA extractions

Genomic DNA was extracted from peripheral blood using a Microlab STAR Liquid Handler (Hamilton, Bonaduz, Switzerland) integrated with a Chemagen automated DNA extraction system (Chemagen AG, Baesweiler, Germany). The DNA samples were quantified using a Qubit fluorometer (Thermo Fisher Scientific, Waltham, MA, USA).

Genotyping and imputation

Genotyping was performed by using the Affymetrix 6.0 GeneChip kit (Applied Biosystems, Santa Clara, CA, USA). The details on genotyping procedures and subsequent quality control steps have been previously described in detail[9]. The dataset is deposited in dbGAP under the phs000294.v1.p1 accession code.

Imputation was performed using the Michigan Imputation server, on the Genome Reference Consortium Human Build 37 r1.1 2016, using haplotypes generated by the Trans-Omics for Precision Medicine (TOPMed) program (freeze 5). Raw genotype data were initially pre-processed to ensure compatibility with the server requirements, by aligning them to the reference build from 1000 Genomes Project (1000G) and dbSNP (b151) by Plink 1.9[17] and BCFtools[18]. In the imputation step, we applied server options to filter by an imputation of $R2 > 0.3$ and to perform a frequency check against the TOPMed panel. In the post-imputation stage, only high-quality variants with $R2 \geq 0.6$ and minor allele frequency (MAF) ≥ 1% were retained, leaving 7,263,254 variants for subsequent analyses.

Whole-exome sequencing

Whole-exome sequencing (WES) had been previously performed in this cohort[9] and these data were utilized to ascertain the presence or absence of rare variants associated with familial hypercholesterolemia. Briefly, libraries were prepared using v3 or v4 Sequencing-by-Synthesis kits (Illumina, Inc., San Diego, CA, USA). All samples were sequenced as paired end 76 nucleotide reads on Genome Analyzer II (Illumina). Sequencing reads were mapped using the Burrows-Wheeler Aligner (BWA, v0.7.15)[19] and duplicated reads, either due to polymerase chain reaction (PCR) or optical duplication, were removed using Picard MarkDuplicates (https://broadinstitute.github.io/picard/). All exomes and their flanking 50 base pairs of intronic sequence were considered for this analysis. Before calling germline variants, the quality score of bases in known sites of variation was recalibrated with GATK using dbSNP138 as reference. Cram files were then exported using Samtools (http://www.htslib.org/) and for each sample, the recalibrated BAM mapping file was used to perform local de-novo assembly of haplotypes using GATK HaplotypeCaller and identify germline variants. The variants were then processed and annotated using bcftools, and Ensembl Variant Effector Predictor[20]. Only the variants passing quality filters (Fisher strand<60; quality by depth>2; phred-scaled genotype<20; genotype quality>20; mapping quality>40; read depth>5) were retained for further analysis.

Polygenic score calculation

A coronary artery disease PGS, the metaGRS[2], was calculated. Details on the construction of this score have been previously published, and the weight file (PGS000018) was obtained from the PGS Catalog[21]. Briefly, imputed genotypes for all study participants were aligned with genotypes from a population reference panel (1000 Genomes, 1000G)[22]. The PGS includes 1,745,179 variants, of which 77.4% (1,350,683) were available in the study cohort and reference panel.

The PGS was calculated using the PGS Catalog's *pgsc_calc* pipeline[21] (https://github.com/PGScatalog/pgsc_calc). PGSs were calculated as a weighted sum of allele counts and effect sizes and normalized to have mean 0 and unit variance by adjusting the PGS for genetic ancestry using the loadings of a principal components (PC) analysis derived from the reference panel[23].

Because the distribution of PGS among study patients was expected to differ substantially from that of the general population, standardization was performed with reference to an external population using the *pgsc_calc* pipeline. First, the genetic similarity of study participants to populations in a reference dataset (1000G) was calculated using principal components analysis (PCA). Next, the study participants' PGSs were adjusted according to PCA loadings of all 1000G individuals, and the mean and variance normalised. Additional details regarding this pipeline can be found in the relevant publications[21-22]. Overall, this pipeline produces externally generalisable standardized scores for all included patients, which will be henceforth referred to simply as PGS.

Definition of monogenic familial hypercholesterolaemia

The presence of familial hypercholesterolemia (FH) was ascertained based on the Genomics England PanelApp recommendations, consulted in November 2023[24] [16]. Where a variant associated with FH was found in one of the autosomal dominant gene regions of interest (*APOB, APOE LDLR, LDLRAP1, PCSK9*) or two variants in one of the autosomal recessive gene regions of interest (*ABCG5, ABCG8, CYP27A1, LDLRAP1, LIPA*), it was considered pathogenic if it met the following criteria for deleteriousness:

1) Loss-of-function mutation, defined as nonsense mutations, insertions or deletions, and splicing mutations in *ABCG5, ABCG8, APOE, CYP27A1, LDLR, LDLRAP1,* or *LIPA*;
2) Gain-of-function mutation in *PCSK9*;
3) Missense variant in *ABCG5, ABCG8, APOB, APOE, CYP27A1, LDLR, LDLRAP1,* or *LIPA* with Combined Annotation Dependent Depletion (CADD) score ≥20; or
4) Missense variant in *ABCG5, ABCG8, APOB, APOE, CYP27A1, LDLR, LDLRAP1,* or *LIPA* with CADD score < 20 but denoted 'pathogenic' or 'likely pathogenic' in the ClinVar database (https://www.ncbi.nlm.nih.gov/clinvar/) for hypercholesterolemia.

Clinical endpoints

The patients were followed up for any subsequent cardiovascular events for a median of 19.9 years from study enrolment. Follow-up was done by means of scheduled outpatient visits and standardized telephone contacts. Follow-up by means of visits was attempted in all patients but, when this was not possible, their primary care physicians were contacted. If this was unsuccessful or not possible, a member of the family was contacted. The relevant medical record(s) of the patients reporting an event were obtained for verification purposes, and source data verification was applied to all cardiovascular events.

The primary endpoint of the study was the composite of cardiovascular death, the first recurrence of non-fatal MI, first coronary revascularization through percutaneous intervention or coronary artery bypass graft, and the first occurrence of non-fatal stroke. All events were adjudicated by a Clinical Event Committee (CEC) consisting of

two cardiologists who did not have access to the genotyping results; in the case of disagreement, the opinion of a third cardiologist was required.

Cardiovascular death

All reported deaths were recorded and adjudicated by means of death certificates. Cardiovascular death was defined as death due to cardiovascular causes and included sudden cardiac death, death due to acute myocardial infarction, death due to heart failure, death due to a cerebrovascular event, death due to other cardiovascular causes (*i.e.*, pulmonary embolism, aortic disease, cardiovascular intervention)

Non-fatal myocardial re-infarction

Myocardial re-infarction was defined as follows:

1. Detection of rise and/or fall in cardiac biomarkers with at least one value above the 99th percentile of the upper reference limit together with evidence of myocardial ischemia with at least one of the following:
   - Symptoms of ischemia;
   - ECG changes indicative of new ischemia (new ST-T changes or new left bundle branch block);
   - Development of pathological ECG Q waves;
   - Imaging evidence of a new loss of viable myocardium or a new regional wall motion abnormality.
2. If the event occurred within the context of percutaneous coronary intervention, MI was defined as an increase in cTn values to more than five times the 99th percentile of the upper limit of normal in patients with normal baseline values. In patients with high pre-procedural cTn levels in whom the cTn level is stable ($\leq$20%

variation) or falling, the post-procedural cTn level must increase by >20%. However, the absolute post-procedural value must still be at least five times the 99th percentile of the upper limit of normal. In addition, one of the following elements was required:

- New ischemic ECG changes;
- The development of new pathological Q waves;
- Imaging evidence of new loss of viable myocardium or new regional wall motion abnormality in a pattern consistent with an ischemic etiology;
- Angiographic findings consistent with a procedural flow-limiting complication such as coronary dissection, the occlusion of a major epicardial artery or a side branch occlusion/thrombus, the disruption of collateral flow, or distal embolisation.

3. If the event occurred within the context of coronary artery bypass graft surgery (CABG), MI was defined as an increase in cTn levels to >10 times the 99th percentile of the upper limit of normal in patients with normal baseline cTn values. In patients with high pre-procedural cTn levels in whom the cTn level is stable (≤20% variation) or falling, the post-procedural cTn level must increase by >20%. However, the absolute post-procedural value must still be >10 times the 99th percentile of the upper limit of normal. In addition, one of the following elements is required:

- The development of new pathological Q waves
- An angiographically documented new graft occlusion or new native coronary artery occlusion
- Imaging evidence of a new loss of viable myocardium or a new regional wall

motion abnormality in a pattern consistent with an ischemic etiology.

4. Pathological findings of an acute myocardial infarction

Stroke

Stroke was defined as an acute episode of neurological dysfunction attributable to a central nervous system vascular cause. Stroke had to be documented by imaging (computed tomography scan or magnetic resonance imaging scan) or autoptic evidence. For the primary endpoint, we only considered ischemic stroke, defined as an acute episode of focal brain, spinal, or visual dysfunction caused by an infarction of central nervous system tissue and documented by imaging.

Revascularization

Revascularization was defined as hospitalization for coronary angioplasty with or without stent implantation or CABG. This was defined as any hospital admission (excluding an admission because of myocardial infarction) that ended in revascularisation by means of a percutaneous coronary intervention or coronary artery by-pass graft surgery.

Statistical analysis

Statistical analysis was performed using the R version 4.2.2[25]. For the purpose of descriptive statistics, patients were categorized into low (PGS < 20th percentile), medium (20th percentile <= PGS < 80th percentile) and high (PGS >= 80th percentile) genetic risk categories. Descriptive statistics are reported across PGS categories as means and standard deviations for continuous variables, and as frequencies and percentages for categorical or binary variables. Cumulative incidence of recurrent events by PGS category was depicted using Kaplan-Meier curves.

Body mass index (BMI) was missing in 31 (1.90%) patients, triglyceride levels were

missing in 81 (4.85%) patients, cholesterol levels were missing in 78 (4.67%) patients, and left ventricular ejection fraction was missing in 531 (31.8%) patients.

Cox proportional hazards models were then fitted to assess the association of clinical variables, PGS, as a standardized continuous variable, and monogenic variants with study outcomes after adjustment for age, sex, and 10 genetic PCs to account for population stratification. Proportional hazards assumptions were tested by assessment of Schoenfeld's residuals and Martingale residuals plots were produced for the main models. Sequential models were fitted including:

(1) all clinical variables together ('clinical model'); including smoking, type 1 or 2 diabetes, hypertension, hyperlipidemia, family history of premature coronary artery disease, and secondary prevention medication initiated at index event (beta-blocker, ACE-inhibitor, statin, aspirin and clopidogrel);
(2) all clinical variables together and PGS ('clinical and polygenic model'), including all clinical variables with the addition of the PGS as a standardized continuous variable, and finally;
(3) all clinical variables, PGS and monogenic variants ('clinical, polygenic and monogenic model'), including all clinical variables, the PGS, and a variable indicating presence or absence of monogenic FH.

Hazards were then predicted using these three models, through a 10-fold cross-validation approach. Harrell's C-index for the cross-validated Cox models were computed using the *survcomp* package. Formal comparisons were conducted using the *cindex.comp* function within *survcomp*, employing a dependent samples t-test[26]. In addition to this, the continuous net reclassification index (NRI) was computed with a cutoff time of 20 years, using the *nricens*[27-29] [19–21] package. The time-dependent area

under the receiver operator curve (AUC) was calculated for each of the three sequential models and compared at regular 2-year intervals using the *timeROC*[30] package. For C-indices, the NRI, and the time-dependent AUC, the following comparisons were made:

(1) The 'clinical and polygenic model' *versus* the 'clinical model'; and

(2) The 'clinical, polygenic and monogenic model' *versus* the 'clinical model'.

Sensitivity analyses

In the first sensitivity analysis, a cross-sectional model was built on occurrence of any recurrent event within follow-up to enable and plotting of the more conventional AUC and calculate the continuous net reclassification index (NRI). For this purpose, logistic regression models were built mirroring those of the longitudinal analysis: a 'clinical model', a 'clinical and polygenic model' a 'clinical, polygenic and monogenic model. These models were used to generate predicted risk of recurrence using 10-fold cross validation. Similar to the longitudinal analysis, the AUC for each of the three sequential models was plotted, calculated and formally compared using the *pROC*[31] package for 'clinical and polygenic model' *versus* the 'clinical model'; and for the 'clinical, polygenic and monogenic model' *versus* the 'clinical model'. In addition to this, we aimed to calculate the continuous NRI as a measure of reclassification efficacy using the *PredictABE*[32] package.

In the second sensitivity analysis, we repeated the primary longitudinal analysis on the modified composite outcome of recurrent MI or cardiovascular death only; thus, excluding ischaemic stroke, and revascularization endpoints.

In the third sensitivity analysis, we repeated the primary longitudinal analysis replacing the binary variables of obesity and hypercholesterolaemia with their continuous counterparts, BMI and non-HDL cholesterol. For the purpose of this analysis, missing data was imputed to the cohort mean for 31 (1.90%) patients for BMI, and 78 (4.67%) patients for non-HDL cholesterol.

In a fourth sensitivity analysis, an enhanced clinical prediction model was built including the additional variables of left ventricular ejection fraction during index admission, Syntax score, and type of primary event (STEMI *versus* NSTEMI). A total

of 531 (31.8%) patients were missing data regarding ejection fraction. A complete case analysis approach was used for this sensitivity analysis. The additional value of polygenic and monogenic markers in addition to the enhanced clinical model was evaluated using the same methods as the main analysis.

In the fifth sensitivity analysis, we repeated the primary longitudinal analysis but additionally accounting for competing risks from non-cardiovascular mortality using Fine and Gray models. The additional value of polygenic and monogenic markers in addition to the clinical model accounting for competing risks was evaluated using the same methods as the main analysis.

In the sixth sensitivity analysis, we repeated the primary longitudinal analysis utilising a different PGS, the 1,296,172-SNP multi-ancestry GPS_{Mult} score recently published by Patel *et al.*[33].

In the seventh and final sensitivity analysis, we repeated the primary longitudinal analysis separately for males and females.

Supplemental Results

Sensitivity analyses

In logistic regression models, after adjustment for age, sex and 10 PCs, a 1-SD higher PGS was associated with 31% greater odds of recurrent events (OR 1.31, 95%CI 1.16-1.47, $p=5.58 \times 10^{-6}$). As presented in **Supplementary Figure 3A**, the predictive performance of clinical factors alone (AUC C 0.770 [95%CI 0.748 − 0.794]) was improved by addition of the PGS (AUC C 0.777 [95%CI 0.754 − 0.799]; p-value compared to clinical model = 0.041), with a continuous NRI of 0.158 indicating a positive influence on reclassification. The addition of the PGS and monogenic variants did not lead to significant improvement compared to the model with clinical factors alone (AUC 0.775 [95%CI 0.752 − 0.798]; p-value compared to clinical model = 0.120), with a continuous NRI of 0.113.

The second sensitivity analysis on the restricted outcome of recurrent MI or cardiovascular death yielded similar results to the main analysis. The discriminative ability of the model combining all clinical characteristics (C-index 0.68, 95%CI 0.66-0.70) was improved after addition of the PGS (C-index 0.69, 95%CI 0.66-0.71, p-value compared to clinical model =0.021) and by addition of both the PGS and monogenic variants (C-index 0.68, 95%CI 0.66-0.71, p-value compared to clinical model =0.090), as displayed in **Supplementary Figure 3B.**

The third sensitivity analysis replacing the binary variables of obesity and hypercholesterolaemia with BMI and non-HDL cholesterol also yielded similar results the main analysis. The discriminative ability of the model combining all clinical characteristics (C-index 0.69, 95%CI 0.67-0.61) was improved after addition of the PGS (C-index 0.69, 95%CI 0.68-0.71, p-value compared to clinical model =0.007) and

by addition of both the PGS and monogenic variants (C-index 0.69, 95%CI 0.68-0.71, p-value compared to clinical model =0.014), as displayed in **Supplementary Figure 3C.**

In the fourth sensitivity analysis using an enhanced clinical prediction model including the additional variables of left ventricular ejection fraction, Syntax score and index event type, the discriminative ability of the model combining all enhanced clinical characteristics (C-index 0.71, 95%CI 0.69-0.73) was significantly improved after addition of the PGS (C-index 0.71, 95%CI 0.69-0.73, p-value compared to clinical model=0.045) and both the PGS and monogenic variants (C-index 0.71, 95%CI 0.69-0.73, p-value compared to clinical model =0.016), as displayed in **Supplementary Figure 3D.**

In the fifth sensitivity analysis accounting for competing risks of non-cardiovascular death, the results were very similar to the main analysis. The discriminative ability of the model combining all clinical characteristics (C-index 0.69, 95%CI 0.67-0.71) was improved after addition of the PGS (C-index 0.70, 95%CI 0.68-0.71, p-value compared to clinical model =0.010) and by addition of both the PGS and monogenic variants (C-index 0.69, 95%CI 0.68-0.71, p-value compared to clinical model=0.030), as displayed in **Supplementary Figure 3E.**

The sixth sensitivity analysis using a different coronary artery disease polygenic score, the GPS_{Mult}, also yielded similar results. The discriminative ability of the model combining all clinical characteristics (C-index 0.69, 95%CI 0.67-0.71) was improved after addition of the PGS (C-index 0.69, 95%CI 0.67-0.71, p-value compared to clinical model =0.011) and by addition of both the PGS and monogenic variants (C-index 0.69, 95%CI 0.67-0.71, p-value compared to clinical model=0.035), as displayed in **Supplementary Figure 3F.**

In the seventh and final sensitivity analysis, we performed the main analysis separately in males and females. In men, the discriminative ability of the model combining all clinical characteristics (C-index 0.68, 95%CI 0.66-0.69) was improved after addition of the PGS (C-index 0.68, 95%CI 0.66-0.60, p-value compared to clinical model =0.009) and by addition of both the PGS and monogenic variants (C-index 0.68, 95%CI 0.66-0.60, p-value compared to clinical model=0.006). This was not observed in females, where the model combining clinical characteristics (C-index 0.68, 95%CI 0.62-0.74) performed similarly after addition of the PGS (C-index 0.68, 95%CI 0.62-0.74, p-value compared to clinical model=0.432) and both the PGS and monogenic variants (C-index 0.68, 95%CI 0.62-0.74, p-value compared to clinical model=0.563), though there was a notable loss of power in this analysis as females only constitute a minority, 11.3%, of the overall cohort. The results are displayed in **Supplementary Figure 5.**

Supplementary Figures

Supplementary Figure 1 – Principal component (PC) analysis plots evaluating the genetic ancestry in the Italian Genetic Study of Early-Onset Myocardial Infarction cohort from the Atherosclerosis, Thrombosis, and Vascular Biology Italian Study Group (ATVB). PC = Principal component, 1000G = 1000 Genomes, AFR = African, AMR = Ad-mixed American, EAS = East Asian, EUR = European, SAS = South Asian.

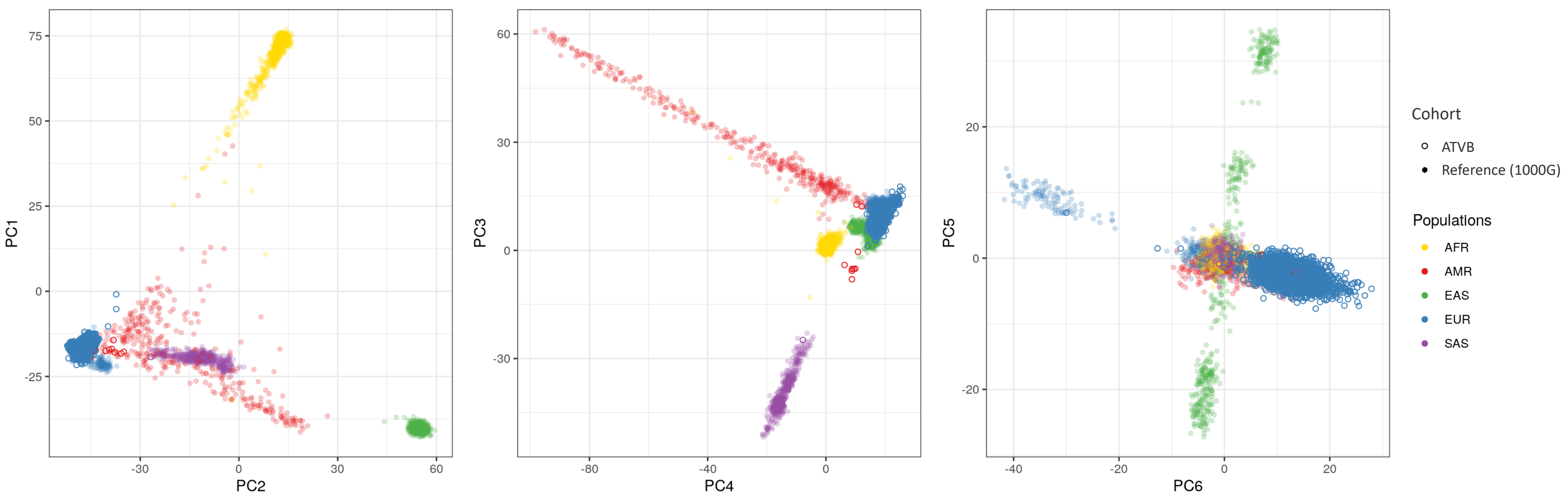

Supplementary Figure 2 – Standardized polygenic score (PGS) distribution among early-onset myocardial infarction patients with (n=846) *versus* without (n=824) recurrent events

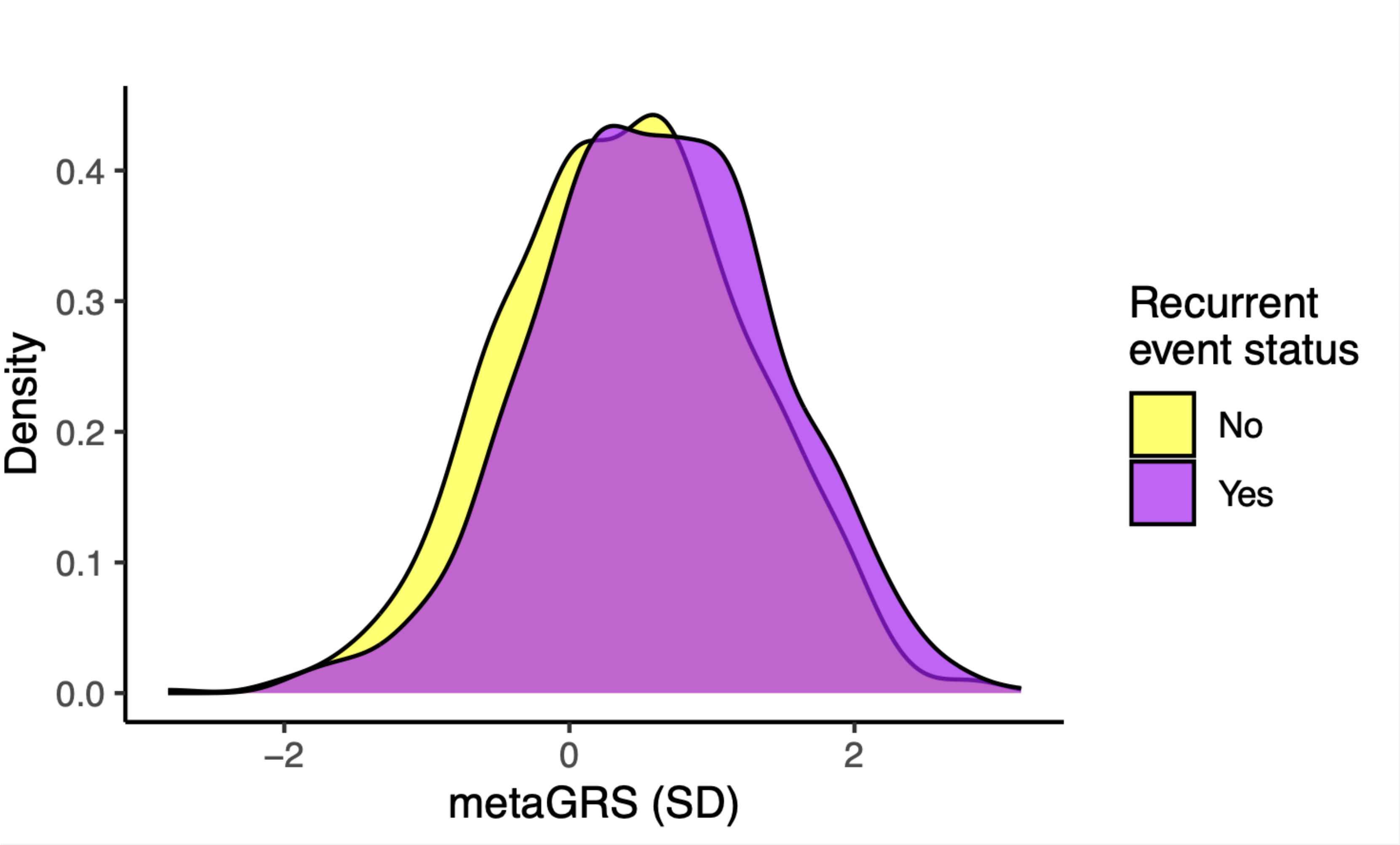

Supplementary Figure 3 – Sensitivity analyses evaluating: **A.** Area under the receiver operator curve (AUC) in logistic regression of recurrent cardiovascular events at 20 years predicted using all clinical factors (blue), clinical factors and polygenic score (purple), and all clinical factors, polygenic score and monogenic variants (dark red). All models include age, sex, and 10 genetic principal components as covariates. **B.** Discrimination ability of conventional risk factors, polygenic score and monogenic variants for the outcome of recurrent myocardial infarction or mortality during follow-up. **C.** Discrimination ability of conventional risk factors, polygenic score and monogenic variants replacing the binary variables of obesity and hypercholesterolaemia with BMI and non-HDL cholesterol. **D.** Discrimination ability of an enhanced set of clinical risk factors, polygenic score and monogenic variants. **E.** Discrimination ability of conventional risk factors, polygenic score and monogenic variants after accounting for competing risks of non-cardiovascular death. **F.** Discrimination ability of conventional risk factors, monogenic variants, and a different, multi-ancestry polygenic score, GPS_{Mult}. AUC = Area under the curve. STEMI = ST-elevation myocardial infarction, NSTEMI = non-ST-elevation myocardial infarction.

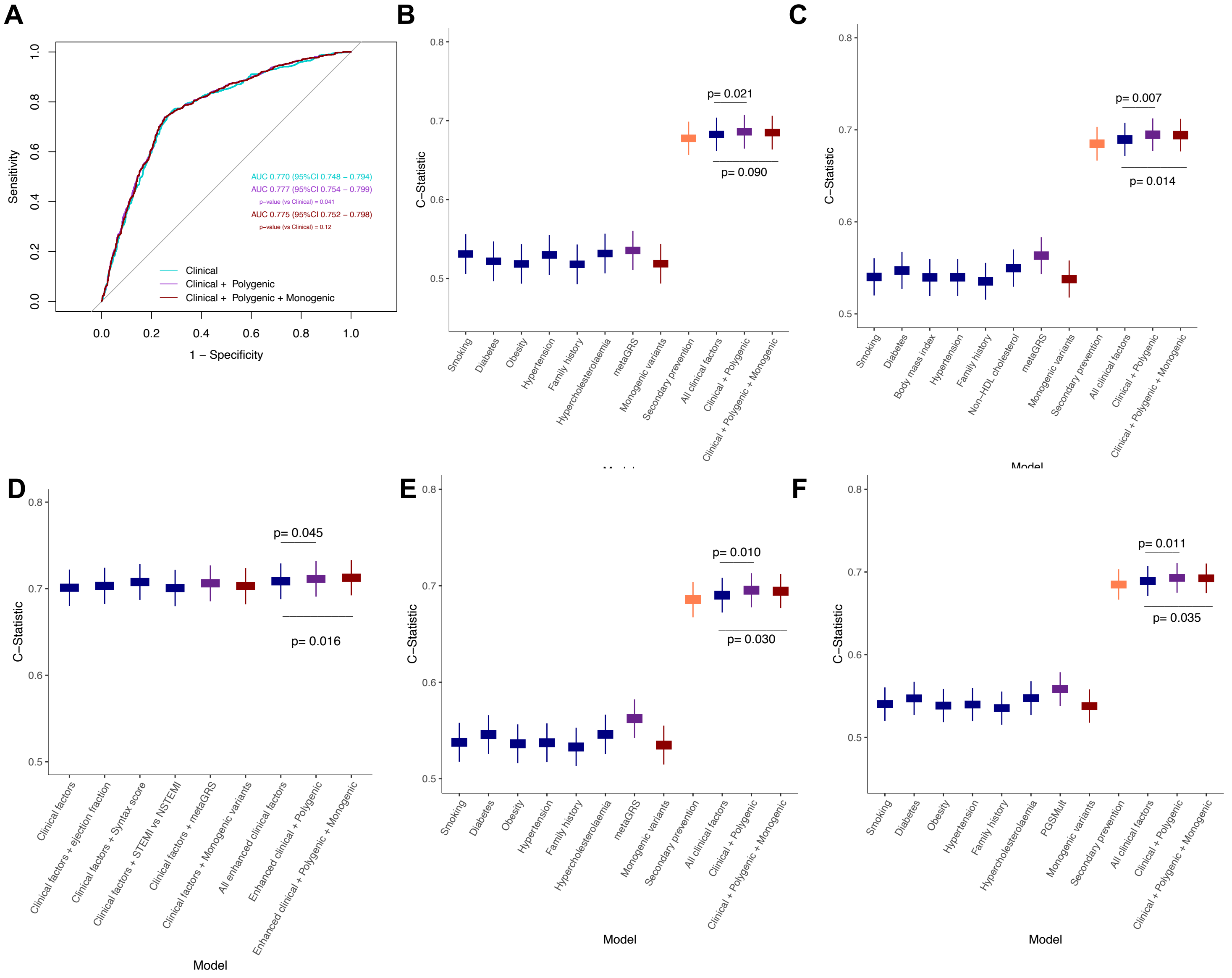
A
B
C
D
E
F
Sensitivity
1 – Specificity
AUC 0.770 (95%CI 0.748 – 0.794)
AUC 0.777 (95%CI 0.754 – 0.799)
p–value (vs Clinical) = 0.041
AUC 0.775 (95%CI 0.752 – 0.798)
p–value (vs Clinical) = 0.12
Clinical
Clinical + Polygenic
Clinical + Polygenic + Monogenic
C–Statistic
Model
p= 0.021
p= 0.090
p= 0.007
p= 0.014
p= 0.045
p= 0.016
p= 0.010
p= 0.030
p= 0.011
p= 0.035
Smoking
Diabetes
Obesity
Body mass index
Hypertension
Family history
Hypercholesterolaemia
Non–HDL cholesterol
metaGRS
PGSMult
Monogenic variants
Secondary prevention
All clinical factors
Clinical + Polygenic
Clinical + Polygenic + Monogenic
Clinical factors
Clinical factors + ejection fraction
Clinical factors + Syntax score
Clinical factors + STEMI vs NSTEMI
Clinical factors + metaGRS
Clinical factors + Monogenic variants
All enhanced clinical factors
Enhanced clinical + Polygenic
Enhanced clinical + Polygenic + Monogenic

Supplementary Figure 4 –Time-dependent area under the receiver operator curve (AUC(t)) for recurrent cardiovascular events over follow-up time predicted using (A) all clinical factors *vs*. all clinical factors and metaGRS, and (B) all clinical factors *vs*. all clinical factors, metaGRS and monogenic variants in Cox regression. All models include age, sex, and 10 genetic principal components as covariates. P-values relate to difference in AUC(t) between the two models shown. AUC(t) = time-dependent rea under the receiver operator curve.

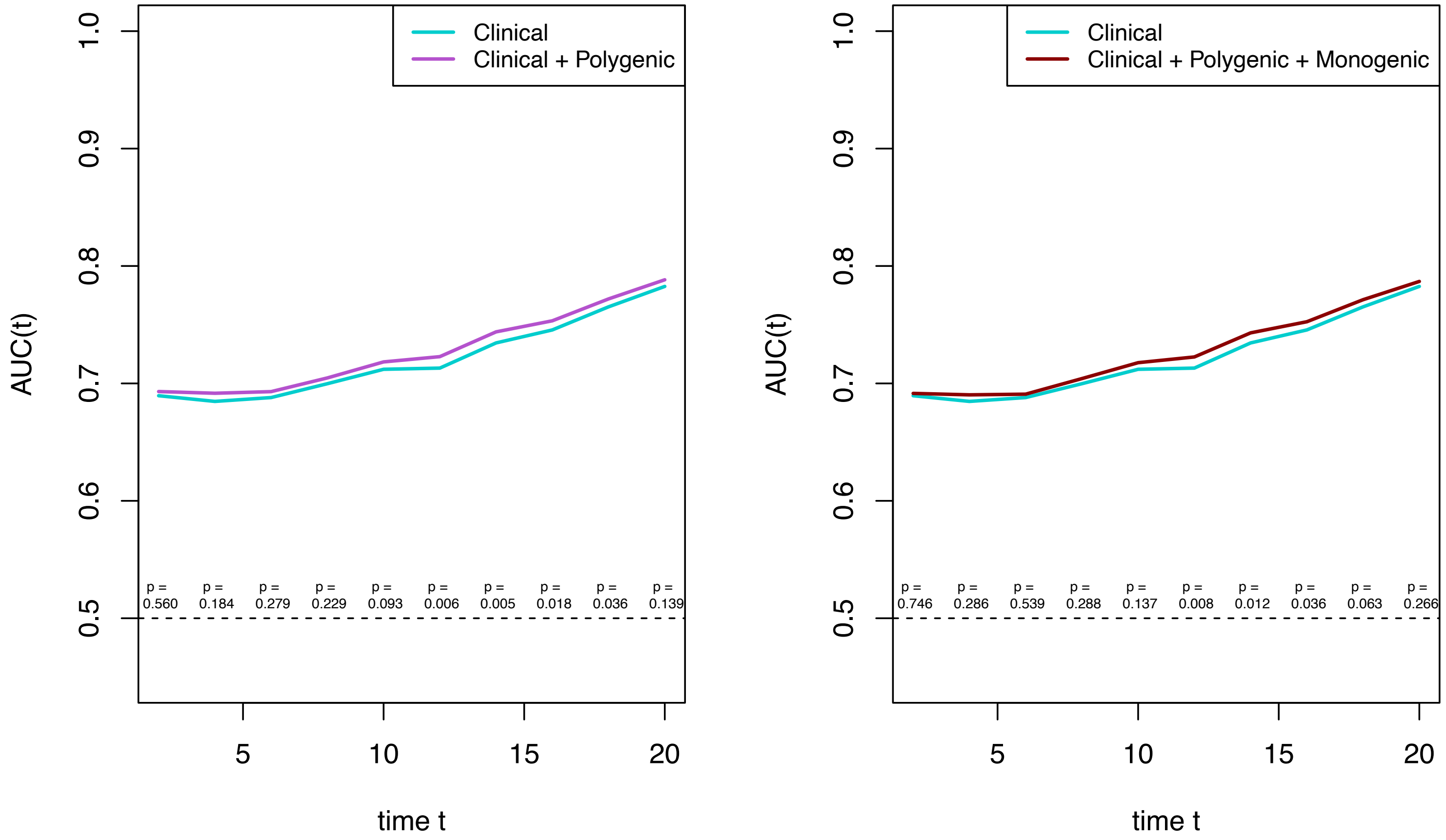
Clinical
Clinical + Polygenic
AUC(t)
1.0
0.9
0.8
0.7
0.6
0.5
p = 0.560
p = 0.184
p = 0.279
p = 0.229
p = 0.093
p = 0.006
p = 0.005
p = 0.018
p = 0.036
p = 0.139
5
10
15
20
time t
Clinical
Clinical + Polygenic + Monogenic
AUC(t)
1.0
0.9
0.8
0.7
0.6
0.5
p = 0.746
p = 0.286
p = 0.539
p = 0.288
p = 0.137
p = 0.008
p = 0.012
p = 0.036
p = 0.063
p = 0.266
5
10
15
20
time t

Supplementary Figure 5 – Sensitivity analyses evaluating: **A.** Discrimination ability of conventional risk factors, polygenic score and monogenic variants for recurrent cardiovascular events in males only. All models include age, sex and 10 genetic principal components as covariates. **B.** Discrimination ability of conventional risk factors, polygenic score and monogenic variants for recurrent cardiovascular events in females only. All models include age and 10 genetic principal components as covariates.

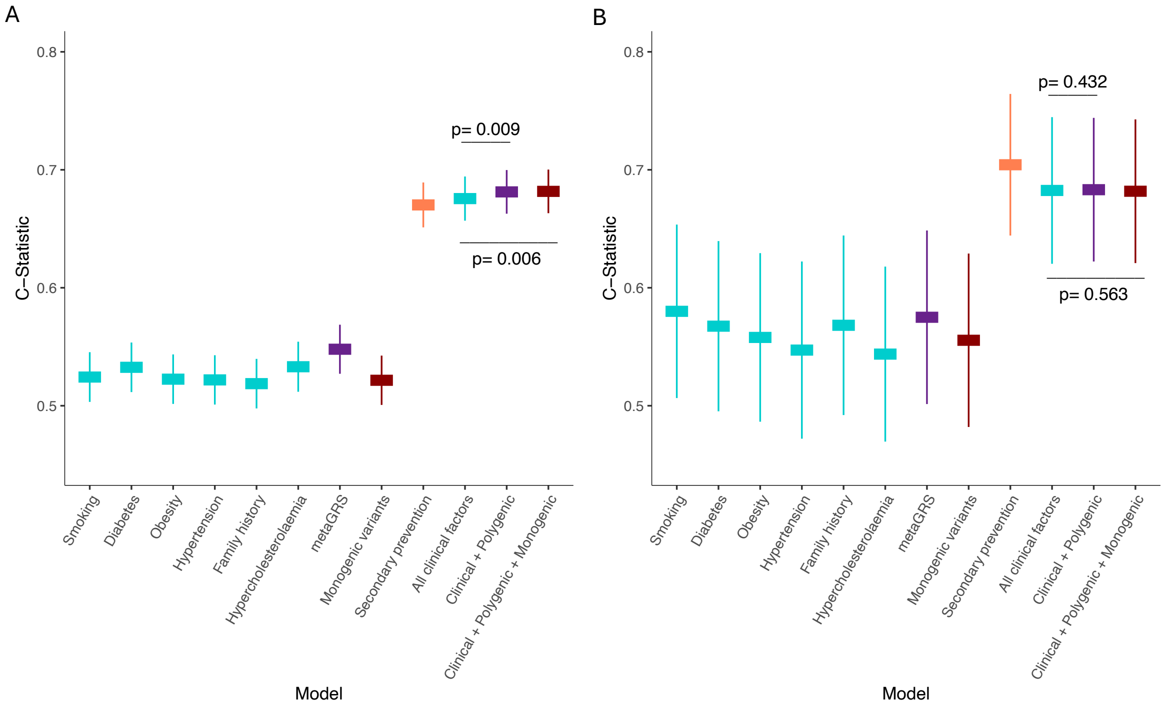

Supplement: Supplementary file 1 [file hcg-17-e004687-s001.pdf]
